# The association between Alu hypomethylation and severity of type 2 diabetes mellitus

**DOI:** 10.1186/s13148-017-0395-6

**Published:** 2017-08-31

**Authors:** Jirapan Thongsroy, Maturada Patchsung, Apiwat Mutirangura

**Affiliations:** 1 School of Medicine, Walailak University, Nakhon Si Thammarat, Thailand; 2 Inter-Department Program of Biomedical Sciences, Faculty of Graduate School, Chulalongkorn University, Bangkok, Thailand; 3 Center for Excellence in Molecular Genetics of Cancer and Human Diseases, Chulalongkorn University, Bangkok, Thailand; 4 Department of Anatomy, Faculty of Medicine, Chulalongkorn University, Bangkok, Thailand

**Keywords:** DNA methylation, Diabetes mellitus, Genomic instability, Alu, Senescence

## Abstract

**Background:**

Cellular senescence due to genomic instability is believed to be one of the mechanisms causing health problems in diabetes mellitus (DM). Low methylation levels of Alu elements or Alu hypomethylation, an epigenomic event causing genomic instability, were commonly found in aging people and patients with aging phenotypes, such as osteoporosis.

**Results:**

We investigate Alu methylation levels of white blood cells of type 2 DM, pre-DM, and control. The DM group possess the lowest Alu methylation (*P* < 0.001, *P* < 0.0001 adjusted age). In the DM group, Alu hypomethylation is directly correlated with high fasting blood sugar, HbA1C, and blood pressure.

**Conclusion:**

Genome-wide hypomethylation may be one of the underlining mechanisms causing genomic instability in type 2 DM. Moreover, Alu methylation levels may be a useful biomarker for monitoring cellular senescence in type 2 DM patients.

**Electronic supplementary material:**

The online version of this article (10.1186/s13148-017-0395-6) contains supplementary material, which is available to authorized users.

## Background

Diabetes mellitus or DM is a complex multifactorial disorder in which the person has high blood glucose (hyperglycemia) [1, 2]. Type 2 DM is the most common type of diabetes. At present, an estimated 387 million people have DM, and that number is expected to reach 592 million by 2035 [3]. DM patients possess increased risk of various geriatric conditions; therefore, type 2 DM is one of the most serious health problems in the world [4–7]. Cellular senescence, particularly vascular senescence, is believed to be a main contributing factor to DM-related complications, such as retinopathy, kidney failure, cerebrovascular disease, and delay wound healing [8, 9]. Nevertheless, the cellular senescence mechanism in DM remains to be explored.

A low Alu methylation level, Alu hypomethylation, has been reported to represent a cellular senescence biomarker [10, 11]. There are more than 1 million copies of Alu elements, a class of short intersperse elements [12]. Alu elements involve up to 11% of the human genome [13]. Alu hypomethylation was first found in white blood cells (WBCs) of aging people during ages 34–68 years [10, 14]. In contrast to aging, higher Alu methylation levels were found in individuals who had catch-up growth during newborn periods [15]. Alu hypomethylation may play a role in the senescence process. When age is adjusted, Alu hypomethylation is independently associated with lower bone mass density including osteopenia and osteoporosis and a high body mass index [11]. Therefore, it is interesting to explore whether cellular senescence of DM also correlates with Alu hypomethylation. Many studies concluded that genome-wide hypomethylation can promote genomic instability [16, 17]. This study will provide information about whether or not global hypomethylation promoting genomic instability is involved in DM pathogenesis.

To investigate Alu methylation changes during type 2 DM development, we measured the level of Alu methylation in normal, pre-type 2 DM, and type 2 DM patients by ALU-Combined Bisulfite Restriction Analysis. COBRA-interspersed repetitive sequence PCR is a highly accurate quantitative methylation measurement. Where pyrosequencing cannot demonstrate DNA methylation pattern, COBRA, detecting more than one CpG site, can (Additional file 1: Figure S1). In many cases, the DNA methylation pattern was more sensitive in revealing DNA methylation changes than DNA methylation levels alone [18–20].

## Methods

### Participants

For the study, we used 240 cases that were being monitored by fasting blood sugar (FBS) levels and were classified into three groups: normal (80 samples), pre-DM (80 samples), and DM (80 samples). Pre-DM and DM were admitted to the Tambon Health Promoting Hospital, Thailand, between 2015 and 2016. Sample ages were between 15 and 80 years. All subjects voluntarily participated in the study. The study was reviewed and approved by the Ethics Clearance Committee on Human Rights Related to Research Involving Human Subjects, Walailak University, Nakhon Si Thammarat, Thailand. Written informed consent was obtained from each participant**.**

### DNA extraction and bisulfite DNA modification

DNA was extracted from buffy coat using proteinase K digestion and phenol-chloroform extraction protocols. Bisulfite treatment was performed as per standard protocols, with some modifications. Briefly, denatured genomic DNA was incubated in 0.22 M NaOH at 37 °C for 10 min, followed by addition of 30 μl of 10 mM hydroquinone and 520 μl of 3 M sodium-bisulfite for 16–20 h at 50 °C. Subsequently, the DNA was purified and incubated in 0.33 M NaOH at 25 °C for 3 min, ethanol precipitated, washed with 70% ethanol, and resuspended in 20 μl of H_2_O.

### ALU-Combined Bisulfite Restriction Analysis (COBRA)

To observe methylation levels of Alu in samples, the sodium-bisulfite-treated DNA in each sample was amplified by PCR containing 1x PCR buffer (Qiagen, Germany), 0.2 mM of deoxynucleotide triphosphate (Promega, USA), 1 mM of magnesium chloride (Qiagen, Germany), 25 U of HotStarTaq DNA Polymerase (Qiagen, Germany), and 0.3 μM primer pairs: ALU-BRev (5′-CTAACTTTTTATATTTTTAATAAAAACRAAATTTCAC CA-3′) where R = A and G and Y = C and T. For Alu amplification, the program was set as follows: 95 °C for 15 min, 40 cycles of 95 °C for 45 s, 57 °C for 45 s, and 72 °C for 45 s, followed by a final extension of 72 °C for 7 min [20]. Alu PCR products were subjected to COBRA using 2 U of TaqI (Thermo scientific, USA), 2 U of TasI (Thermo scientific, USA), 5x NEB3 buffer (New England Biolabs, USA), and 1 μg/ul bovine serum albumin (BSA) (New England Biolabs, USA) and incubated at 65 °C overnight. The cut PCR products were analyzed by 8% acrylamide gel and SYBR stain (Lonza, USA). The band intensity of Alu methylation was observed and measured by typhoon fla 7000 and ImageQuanNT Software (Amersham biosciences, UK) (Additional file 1: Figure S1) [11].

### Methylation analysis

ALU methylation analysis consisted of calculating the band intensity of the five Alu product sizes; 133, 90, 75, 58, and 43 bp according to the formula: *A* = 133/133, *B* = 58/58, *C* = 75/75, *D* = 90/90, *E* = 43/43, and *F* = [(*E* + *B*) − (*C* + *D*)]/2. The Alu methylation level was calculated from [(*E* + *B*) × 100]/(2A + *E* + *B* + *C* + *D*).

### Statistical analyses

Data was analyzed with SPSS statistical software. The average and distributions of characteristic data of type 2 DM are presented as the mean ± SD and median. Pearson’s correlation between the Alu methylation pattern and characteristic data of the population were used to determine 95% confidence intervals of association. *T*-score was used to divide the subjects into three groups: normal, pre-DM, and DM. *T*-test was used to determine the differences using a *P* value threshold of 0.05 between the groups in the matched cases based on fasting blood sugar (FBS). Logistic regression models were used to evaluate the association between FBS by *T*-score as a dependent variable specified as a binary outcome, normal and DM and %mC, %mCmC loci, %uCmC loci, %mCuC loci, and %uCuC loci as independent predictors. All independent variables were used in the same model.

## Results

### Alu methylation in patients with type 2 diabetes

**Fig. 1:**
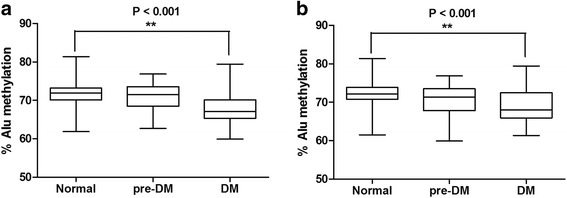
The percentage of each Alu methylation level in normal, pre-DM, and DM samples grouped by fasting blood sugar level (**a**) and hemoglobin A1C (HbA1C) (**b**). Boxes represent interquartile ranges (25th to 75th percentile) and median lines represent the 50th percentile. The whiskers represent minimum and maximum values. *P < 0.05, **P < 0.001 (Mann-Whitney test)

Fasting blood sugar (FBS) levels were used to classify the 240 samples into three groups: 80 normal controls, 80 pre-DM, and 80 type 2 DM. These samples were also classified using HbA1C levels resulting in several patients changing groups (42 normal, 113 pre-DM, and 85 DM) compared to the values from the FBS indicator (Table 1). Next, we used the ALU-Combined Bisulfite Restriction Analysis (COBRA-Alu) to measure Alu methylation in each sample. When comparing Alu methylation levels of the normal with the pre-DM and type 2 DM patients grouped according to FBS, we found that the level of Alu methylation was lowest in type 2 DM (*P* < 0.001) (Fig. 1a). Similarly, when grouped by the HbA1C indicator, we found significantly decreased Alu methylation levels in type 2 DM when compared with normal (*P* < 0.001) (Fig. 1b).

**Table 1:** Sample size, age, and body mass index (BMI) in each group by FBS and HbA1C indicator

	Group			*P* value
	Normal	Pre-DM	DM	
FBS indicator				
*n*	80	80	80	
Sex				
Male	15 (18.75%)	12 (15.00%)	24 (30.00%)	
Female	65 (81.25%)	68 (85.00%)	56 (70.00%)	
Age (years) (mean ± SD)	50.04 ± 9.65	51.21 ± 9.27	59.99 ± 11.88	<0.001
BMI (kg/m^2^) (mean ± SD)	25.04 ± 3.81	25.64 ± 3.97	27.17 ± 9.89	0.0984
HbA1C indicator				
*n*	42	113	85	
Sex				
Male	5 (11.90%)	28 (24.78%)	11 (12.94%)	
Female	37 (88.10%)	85 (75.22%)	74 (87.06%)	
Age (years) (mean ± SD)	47.67 ± 9.03	53.64 ± 10.67	56.89 ± 11.73	<0.001
BMI (kg/m^2^) (mean ± SD)	23.82 ± 3.66	26.45 ± 7.94	26.31 ± 5.07	0.0627

**Fig. 2:**
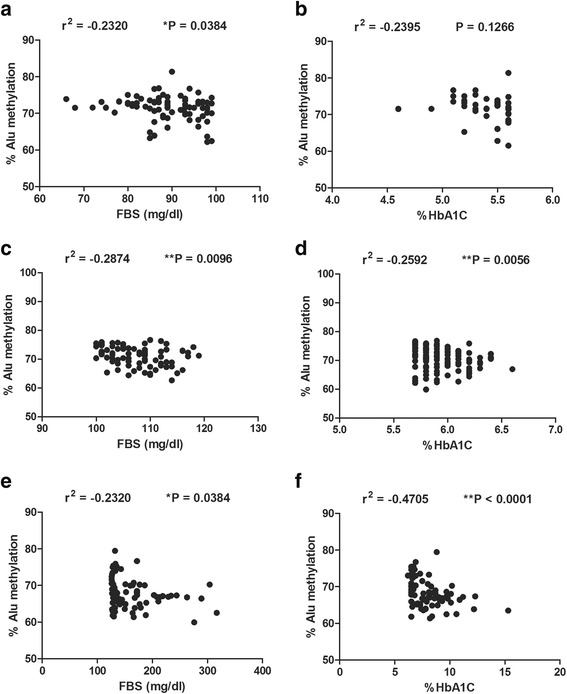
Association between Alu methylation with FBS and HbA1C levels. Correlation between % Alu methylation and FBS in normal (**a**) pre-DM (**b**), and DM (**c**). Correlation between Alu methylation with HbA1C in normal (**d**), pre-DM (**e**), and DM (**f**). Pearson’s correlation coefficients (*r*) with *P* values are indicated (*P < 0.05, **P < 0.001)

Correlations between Alu methylation and FBS or HbA1C in normal, pre-DM, and type 2 DM are reported (Fig. 2 a–f). All groups showed higher biochemical markers of DM, FBS or HbA1C, and lower Alu methylation levels (Fig. 2 a–f). Moreover, the most striking comparison was the correlation between Alu methylation and HbA1C in DM (*r* ^2^ = −0.4705, *P* < 0.0001) (Fig. 2f).

### Age- and sex-adjusted correlation

**Fig. 3:**
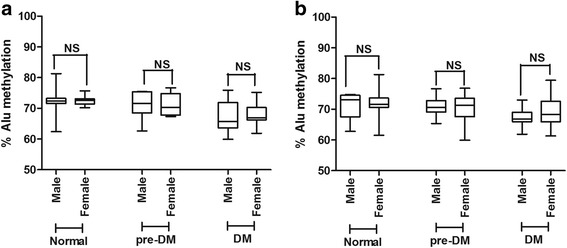
Comparisons of Alu methylation levels between male and female in normal, pre-DM, and DM when grouped using FBS indicator (**a**) and HbA1C indicator (**b**). The boxes represent interquartile ranges (25th to 75th percentile) and median lines represent the 50th percentile. The whiskers represent minimum and maximum values. *P < 0.05, **P < 0.001 (Mann-Whitney test)

To determine if sex influenced Alu methylation levels, significant differences in Alu methylation levels were detected between males and females. Here, we divided males and females into normal, pre-DM, and type 2 DM groups. Alu methylation levels were not significantly different between males and females in either FBS (Fig. 3a) or HbA1C groups (Fig. 3b). This is consistent with previous works that have shown that sex does not affect Alu methylation [10, 15].

**Fig. 4:**
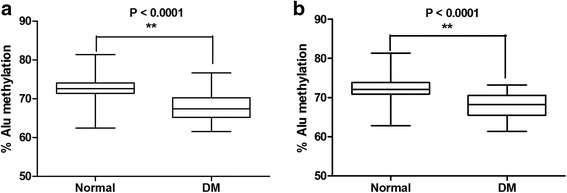
The percentage of Alu methylation in age-matched pairs between normal samples and DM samples in both the FBS (**a**) and HbA1C groups (**b**). The values from 30 independent age-matched pairs are shown as box plots, with the boxes representing interquartile ranges (25th to 75th percentile) and median lines representing the 50th percentile. The whiskers represent minimum and maximum values. *P < 0.05, **P < 0.001 (Mann-Whitney test)

The average age in normal and type 2 DM groups was not the same in our data (Table 1). To adjust for age difference, normal samples were matched to same-age DM samples to produce 30 age-matched pairs. The age-matched pairs showed significantly decreased Alu methylation in DM compared with normal in both the FBS (*P* < 0.001) and HbA1C groups (*P* < 0.001) (Fig. 4 a, b, respectively). Results were similar to findings before age adjustment (Fig. 1a, b).

**Fig. 5:**
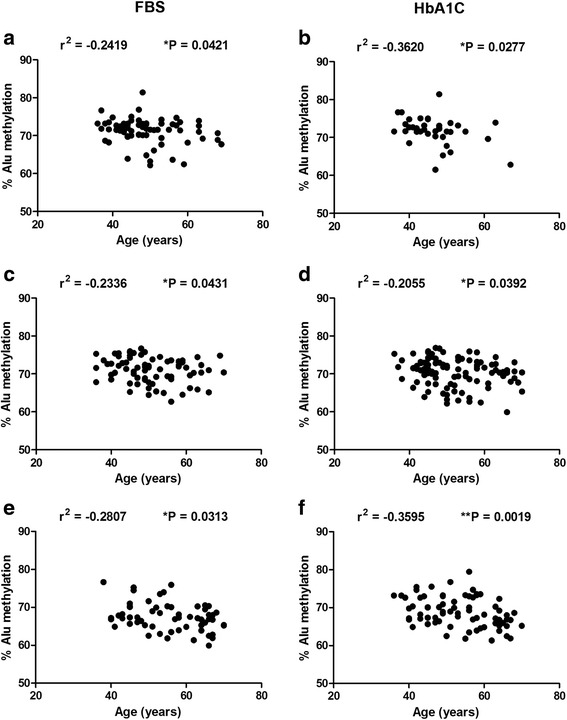
Correlation between Alu methylation and age in the FBS and HbA1C groups. Correlation between % Alu methylation and age in normal (**a**) pre-DM (**b**), and DM (**c**) for the FBS group. Association between Alu methylation with age grouped by HbA1C for normal (**d**), pre-DM (**e**), and DM (**f**). Each plot represents Alu methylation levels of sample. Pearson’s correlation coefficients (*r*) with *P* values are indicated (*P < 0.05, **P < 0.001)

A significant negative correlation between Alu methylation and age was observed in all groups (normal, pre-DM, and DM) when using FBS (Fig. 5a–e) (*r* ^2^ = −0.2419, *P* = 0.0421, *r* ^2^ = −0.2336, *P* = 0.0431, and *r* ^2^ = −0.2807, *P* = 0.0313, respectively) and HbA1C (Fig. 5b–f) (*r* ^2^ = −0.3620, *P* = 0.0277, *r* ^2^ = −0.2055, *P* = 0.0392, and *r* ^2^ = −0.2320, *P* = 0.0019, respectively).

### Correlation between Alu methylation and hypertension in normal, pre-DM, and type 2 DM

**Fig. 6:**
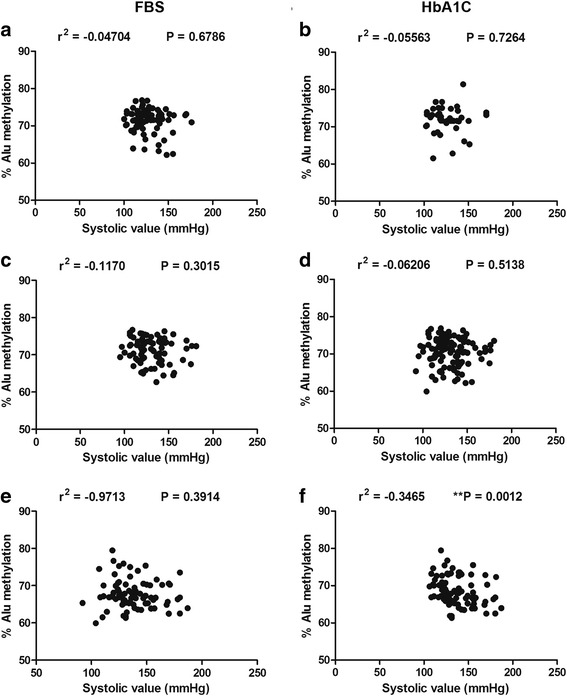
Relationship between Alu methylation and systolic pressure for normal (**a**) pre-DM (**b**), and DM (**c**) samples grouped by FBS. Alu methylation and systolic pressure for normal (**d**), pre-DM (**e**), and DM (**f**) samples grouped by HbA1C. Pearson’s correlation coefficients (*r*) with *P* values are indicated (**P < 0.001)

**Fig. 7:**
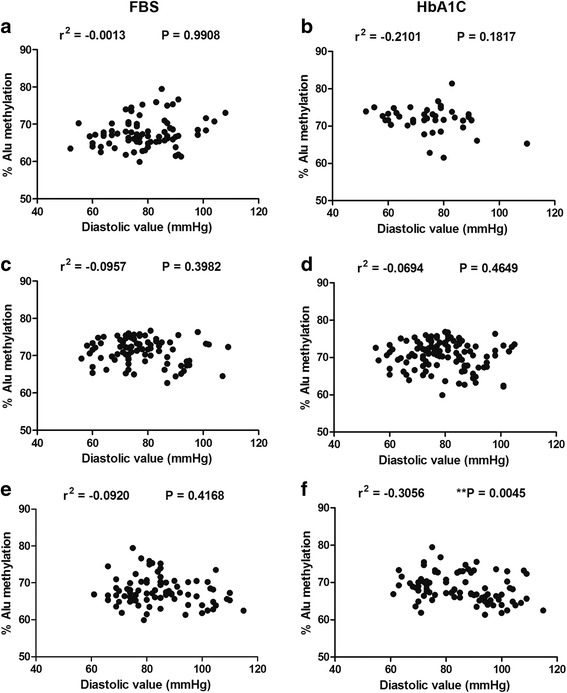
Association between Alu methylation and diastolic pressure for normal (**a**), pre-DM (**b**), and DM (**c**) samples grouped by FBS. Alu methylation and diastolic pressure for normal (**d**), pre-DM (**e**), and DM (**f**) samples grouped by HbA1C. Pearson’s correlation coefficients (*r*) with *P* values are indicated (**P < 0.001)

Hypertension in type 2 DM is believed to be one of the severe consequences of vascular senescence [8, 21, 22]. We investigated the relationship between Alu methylation levels and blood pressure (systolic and diastolic) for FBS and HbA1C groups. The results showed a significantly negative association between Alu methylation and systolic pressure only in type 2 DM patients when classified by HbA1C (Fig. 6f). Intriguingly, a similar negative correlation between Alu methylation and diastolic pressure was observed in only type 2 DM patients for the HbA1C group (Fig. 7f). There was no significant correlation between Alu methylation and hypertension in normal or pre-DM samples in either the FBS (Figs. 6a, c and 7a, c) or HbA1C groups (Figs. 6b, d and 7b, d).

**Fig. 8:**
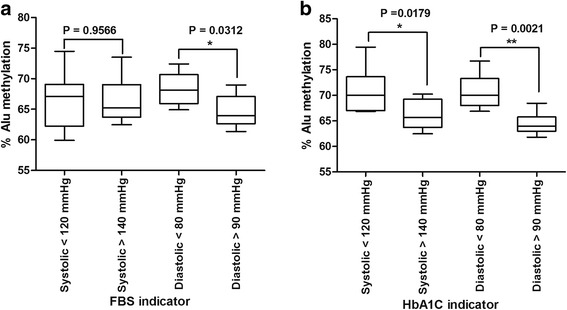
The percentage of Alu methylation levels in type 2 DM between high systolic/diastolic and normal to adjusted for age when grouped using FBS indicator (**a**) and HbA1C indicator (**b**). The boxes represent interquartile ranges (25th to 75th percentile) and median lines represent the 50th percentile. The whiskers represent minimum and maximum values. *P < 0.05, **P < 0.001 (Mann-Whitney test)

To prove that the correlation between Alu hypomethylation and hypertension was not influenced by age, we performed age-adjusted experiments (Fig. 8 a, b). Interestingly, the significant correlation was revealed in the DM using HbA1C as indicator (*P* = 0.0312) (Fig. 8a).

## Discussion

Cellular dysfunction due to aging process was proposed to be one of the pathogenic mechanisms of type 2 DM [8, 9]. One known mechanism of cellular aging is decrement of genome-wide methylation-promoting genomic instability [17, 23]. Therefore, in this study, we proved that Alu methylation in type 2 DM patients was lower than that in the general population. Although we found significant differences between DM phenotype and Alu methylation level, there is a significant overlap between Alu methylation levels of DM cases and controls. Moreover, there is no significant difference between pre-DM and normal controls. These limit the use of using Alu methylation levels directly as a biomarker for clinical application. Further study should be performed to evaluate how the change of Alu methylation level of each individual is correlated with progression of the diseases.

Interestingly, we demonstrated a direct correlation between Alu hypomethylation levels with high blood pressure in DM samples when grouped by HbA1C. This association is similar to the correlation between Alu hypomethylation and lower bone mass [11]. Moreover, the fact that the correlation was found only when grouping DM by HbA1C reflected that the finding was a permanent change. Target cellular defects causing high blood pressure in DM are not WBC. The underlining reason of how Alu hypomethylation in WBC is associated with high blood pressure may be that the mechanism causing Alu hypomethylation in DM is systemic and methylation levels of other tissues remained to be explored. Nevertheless, the fact that Alu hypomethylation in WBC is associated with several geriatric degenerative diseases suggests that Alu hypomethylation levels in white blood cells are a promising biomarker for senescence monitoring.

Mechanisms of aberrant methylation and genomic instability in cellular senescence and type 2 DM are unclear. It is possible that oxidative stress products increase glutathione levels that could influence epigenetic processes including DNA and histone methylation by limiting the availability of S-adenosylmethionine (SAM) leading to DNA hypomethylation [24–26]. Alternatively, the common oxygen radical adduct 8-hydroxyguanine in DNA can dramatically inhibit the process of DNA methylation, so free radical damage may explain the decreased Alu methylation [27–30]. Possibly, high glucose concentration-induced cytosolic Ca2+ and ERK1/2 activation increases mitochondrial fragmentation and reactive oxygen species (ROS) levels [31–33]. Hence, hyperglycemia could promote cellular senescent formation by metabolic changes and tissue damage from oxidative stress resulting in increased free radicals that could lead to genomic hypomethylation and genomic instability in type 2 DM [34].

## Conclusions

Our results not only showed significant Alu hypomethylation levels in type 2 DM but also its association with high blood pressure. Therefore, Alu methylation is a promising biomarker for monitoring type 2 DM complications. Moreover, Alu elements should be considered as epigenome-editing targets for future research in DM disability prevention and treatment.

## Additional file

Additional file 1: Figure S1. Alu methylation patterns of COBRA-Alu assay. (A) The Alu amplicons are 133 bp and contain 2 CpG-dinucleotides. (B) A schematic representation of the COBRA-Alu assay shows the methylation patterns of Alu amplicons, including fully methylated loci (mCmC), unmethylated loci (uCuC) and two partially methylated forms (mCuC and uCmC). (C) For bisulfate treatment, methylated cytosine bases are not changed to uracil bases, whereas the unmethylated cytosine bases are converted to uracil bases. After the PCR products are digested with Taq1 restriction enzyme, the digested products are mCmC (43, 32 and 58 bp), uCuC ( 133 bp), mCuC ( 43 and 90 bp) and uCmC ( 75 and 58 bp). (TIFF 98 kb)

## Abbreviations

COBRA: Combined Bisulfite Restriction Analysis
DM: Diabetes mellitus
FBS: Fasting blood sugar
HbA1C: Hemoglobin A1c
ROS: Reactive oxygen species
SAM: S-adenosylmethionine
WBCs: White blood cells
