# Supplementary figures and images for: The association between Alu hypomethylation and severity of type 2 diabetes mellitus
Source: Clin Epigenetics. 2017 Aug 31;9:93. doi: 10.1186/s13148-017-0395-6 (PMC5580285; doi:10.1186/s13148-017-0395-6)

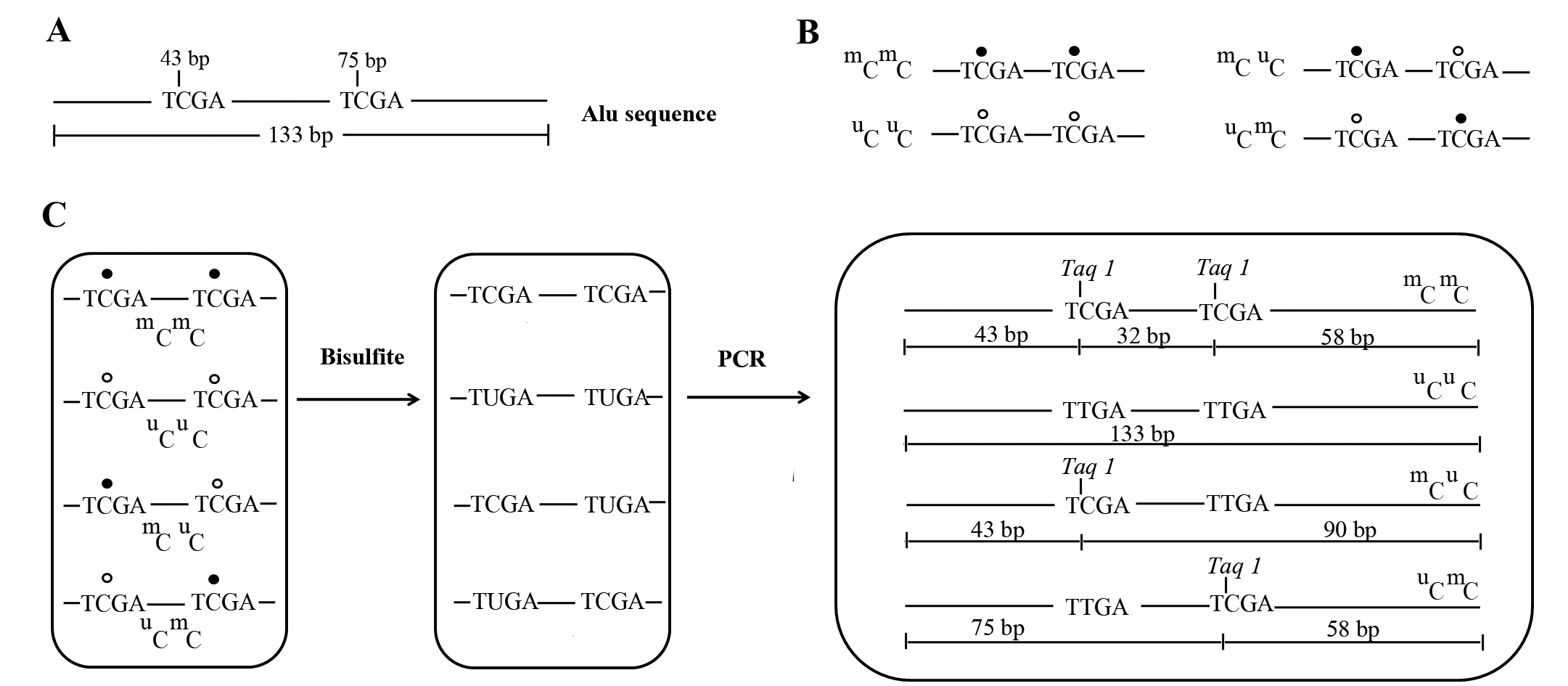

Supplement: Additional file 1: Figure S1. — Alu methylation patterns of COBRA-Alu assay. (A) The Alu amplicons are 133 bp and contain 2 CpG-dinucleotides. (B) A schematic representation of the COBRA-Alu assay shows the methylation patterns of Alu amplicons, including fully methylated loci (mCmC), unmethylated loci (uCuC) and two partially methylated forms (mCuC and uCmC). (C) For bisulfate treatment, methylated cytosine bases are not changed to uracil bases, whereas the unmethylated cytosine bases are converted to uracil bases. After the PCR products are digested with Taq1 restriction enzyme, the digested products are mCmC (43, 32 and 58 bp), uCuC ( 133 bp), mCuC ( 43 and 90 bp) and uCmC ( 75 and 58 bp). (TIFF 98 kb) [file 13148_2017_395_MOESM1_ESM.tif]
